# Indicator-activities to apply primary health care principles in national or large-scale community health worker programs in low-and middle-income countries: a Delphi exercise

**DOI:** 10.1186/s12889-022-13996-y

**Published:** 2022-08-22

**Authors:** Shagufta Perveen, Caroline Laurence, Mohammad Afzal Mahmood

**Affiliations:** 1 School of Public Health, Faculty of Health and Medical Sciences, The University of Adelaide, Level 5 Rundle Mall Plaza, 50 Rundle Mall, Adelaide, South Australia 5000 Australia; 2 Faculty of Medicine, Universitas Airlangga, Surabaya, Indonesia

**Keywords:** Primary health care principles, Community health worker programs, Low-and middle-income countries, Delphi

## Abstract

**Introduction:**

Primary Health Care (PHC) gained considerable momentum in the past four decades and led to improved health outcomes across a wide variety of settings. In low-and middle-income countries (LMICs), national or large-scale Community Health Worker Programs (CHWPs) are considered as vehicles to incorporate PHC principles into healthcare provision and are an essential aspect of the PHC approach to achieve health for all and sustainable development goals. The success of CHWPs is rooted in the application of PHC principles. However, there is evidence that shows patchy implementation of PHC principles across national CHWPs in LMICs. This may reflect the lack of information on what activities would illustrate the application of these principles in CHWPs. This study aimed to identify a set of core/indicator-activities that reflect the application of PHC principles by CHWPs in LMICs.

**Methods:**

A two-round modified Delphi study was undertaken with participants who have extensive experience in planning, implementation and evaluation of CHWPs. Survey design and analysis was guided by the four PHC principles namely Universal Health Coverage, Community Participation, Intersectoral Coordination and Appropriateness. Responses were collected using a secure online survey program (survey monkey). In round one, participants were asked to list ‘core activities’ that would reflect the application of each PHC principle and its sub-attributes and challenges to apply these principles in CHWPs. In round two, participants were asked to select whether they agree or disagree with each of the activities and challenges. Consensus was set a priori at 70% agreement of participants for each question.

**Results:**

Seventeen participants from 15 countries participated in the study. Consensus was reached on 59 activities reflecting the application of PHC principles by CHWPs. Based on participants’ responses, a set of 29 indicator-activities for the four PHC principles was developed with examples for each indicator-activity.

**Conclusion:**

These indicator-activities may provide guidance on how PHC principles can be implemented in CHWPs. They can be used in the development and evaluation of CHWPs, particularly in their application of PHC principles. Future research may focus on testing the utility of indicator-activities on CHWPs in LMICs.

**Supplementary Information:**

The online version contains supplementary material available at 10.1186/s12889-022-13996-y.

## Background

Primary Health Care (PHC) as an approach to achieve ‘health for all’ implies that all people, everywhere, deserve the right care [1]. In the context of many low- and middle-income countries (LMICs), the health systems are fragile and not adequately strengthened in terms of infrastructure and resources, limiting their capacity to reach out to the whole population to achieve ‘health for all’. Therefore, Community Health Worker Programs (CHWPs) are considered as an essential aspect of the PHC approach to achieve health for all and sustainable development goals in LMICs [2]. As part of the PHC approach, CHWPs aim to reach wider population at their doorstep [3, 4]. The foundation of CHWPs was based on PHC principles in order to achieve improvements in health outcomes [5–8]. However, the process of implementing PHC principles in general has been challenging [9]. Lack of PHC integration has been identified as one of the main limits to programs’ efficacy in LMICs [10]. Lack of uniformity in the application of PHC principles is also evident in national CHWPs in LMICs particularly for the principles of intersectoral coordination and appropriateness [11, 12]. This may be because it is difficult to define what the application of the PHC principles in a CHWP would look like, and that may be due to the lack of well-defined indicators or the types of activities that may represent the application of PHC principles.

There are various frameworks and indicators available which are focused on assessing the practice and performance of CHWs [13]. Some examples include the CHW Common Indicators Project (CIP), CHW Assessment and Improvement Matrix (AIM), Accompanimeter 1.0’ tool and 5-SPICE framework.

The CHW-CIP proposes a set of common process and outcome constructs and indicators, such as workers’ roles, support and supervision for workers, health and social needs and self-reported health status of participants to assess CHW practice and program implementation [14]. The ‘Accompanimeter 1.0’ tool and 5-SPICE framework developed by Partners in Health (PIH) in the United States focus on programmatic aspects such as workers skill development, incentives, supervision and partnering [15, 16]. The CHW-AIM developed by the USAID-funded Health Care Improvement (HCI) project encompasses various programmatic components which are critical to support CHWs and functionality indicators such as accreditation, supervision and how a community supports a program [17]. Another example is a framework for monitoring the performance of CHWs in LMICs developed by the Frontline Health project [18]. These examples indicate that majority of the frameworks are about processes and functions of the CHWPs and not about the application of PHC principles [13].

With reference to PHC, important initiatives also exist such as the Primary Health Care Performance Initiative (PHCPI), partnership that brings together country policymakers, health system managers, advocates and other development partners to catalyze PHC improvements in LMICs through better measurement, knowledge-sharing, and deploying data for improvement [19]. The measurement, however, focusses on inputs such as facilities and staff, service delivery such as perceived barriers to cost and treatment success rates and outputs such as antenatal care and immunization coverage. The above description highlights that there are important and useful tools to measure programmatic inputs and functionality, however they do not focus on the application of PHC principles.

The 2020 WHO’s operational framework for PHC targets national government leaders in order to strengthen health systems and support countries in scaling up national implementation efforts on PHC [20]. It mentions that a commitment to PHC is founded on the principles of Declaration of Alma Ata and that the approach to PHC includes integrated services, community empowerment and intersectoral policy. The framework is about strategic and operational levers such as political commitment, funding, workforce etc. It encompasses all PHC principles but focuses on PHC implementation efforts at a high level than program level. 

In order to address this gap, clear and carefully chosen indicator-activities are needed that reflect the application PHC principles and will contribute further to the success of CHWPs. Hence, this study aims to identify a set of indicator-activities that reflect the application of the PHC principles by national or large-scale CHWPs in LMICs.

## Methods

### Study design

A two-round modified Delphi study was undertaken to establish consensus on the importance of PHC principles and the core activities reflecting their application in the CHWPs in LMICs. The Delphi technique is an iterative multistage research method where sequential surveys or questionnaires are used to gather individual expert opinion via a number of rounds, as a means of establishing consensus opinion across the group of participants [21, 22]. The benefits of Delphi include the ability to gain the perspectives of a broadly experienced group of individuals and build consensus in an area where relevant literature or evidence may be lacking [21].

### Recruitment of study participants

Participants were recruited using purposive sampling which focused on the recruitment of experts with multi-level perspectives and real-life implementation and evaluation experience rather than a large sample size. This was to ensure that consensus would be grounded in an applied understanding of CHWP implementation and evaluation in LMICs. Selection criteria included: five or more years of experience with national or large-scale CHWPs, in planning, implementation and/or evaluation in LMICs; and also fluent in reading and writing of English language. The selection criteria was not based on the participant’s country of residence. A list of potential participants was devised based on the professional contacts of the research team and a review of the authors of reports and publications related to CHWPs. Recruitment emails were then sent to these potential participants, which included short introductory letter outlining the study’s background and selection criteria, and the ‘informed consent’ form. Overall, 48 potential participants from Afghanistan, Bangladesh, Brazil, Canada, Ethiopia, Ghana, India, Iran, Jordan, Kenya, Malawi, Mozambique, Myanmar, Nepal, Pakistan, Rwanda, South Africa, United Kingdom, Uganda, USA and Zambia were contacted. Twenty-eight individuals responded out of which 20 consented to participate in the study.

### Survey design and development

In this study, survey development, data collection, analysis and reporting of results were guided by the four foundational PHC principles namely universal health coverage (UHC), community participation, intersectoral coordination and appropriateness [5, 23].

#### Operational definition of UHC

It is important to note here that the concept of UHC combines the two early concepts of equity and access for all (universal coverage) and comprehensiveness [5] in its recent definition as “all individuals and communities receive the health services they need – including promotive, protective, preventive, curative, rehabilitative and palliative – of sufficient quality, without experiencing financial hardships [24].”

Use of the PHC principles for the survey structure aimed to facilitate greater participant understanding and a systemic approach to analysis across both survey rounds. National or large-scale CHWPs have been selected for the purpose of understanding the application of PHC principles however, the application is not confined to these programs alone.

#### Round one

A semi-structured qualitative questionnaire was designed for round one. Participants were asked to rate and rank the importance of incorporating each PHC principle in the implementation of national or large-scale CHWPs in LMICs. Participants were also asked to list core activities that would reflect the application of each PHC principle and its sub-attributes (Table 1) and challenges to apply these principles in CHWPs.

**Table 1:** Primary health care principles and their sub-attributes

PHC Principle	Sub-Attributes
Universal Health Coverage	Equity Access Comprehensiveness
Community Participation	-
Intersectoral Coordination	-
Appropriateness	Effectiveness Cultural acceptability Affordability Manageability

#### Round two

In the subsequent second round of the Delphi survey, participants were provided with a summary of the responses from the first round for the purpose of rating, ranking and identifying the core activities that may represent the application of each PHC principle and its sub-attributes along with the challenges for implementing these principles. For the activities and challenges, participants were asked to select whether they ‘agree’ or ‘disagree’ with each of the activities and challenges for the application of PHC principles in CHWPs. An open text box allowing for additional comments was also included with each question. To maintain the privacy and confidentiality of the participants, all responses were de-identified.

### Data collection

**Fig. 1:**
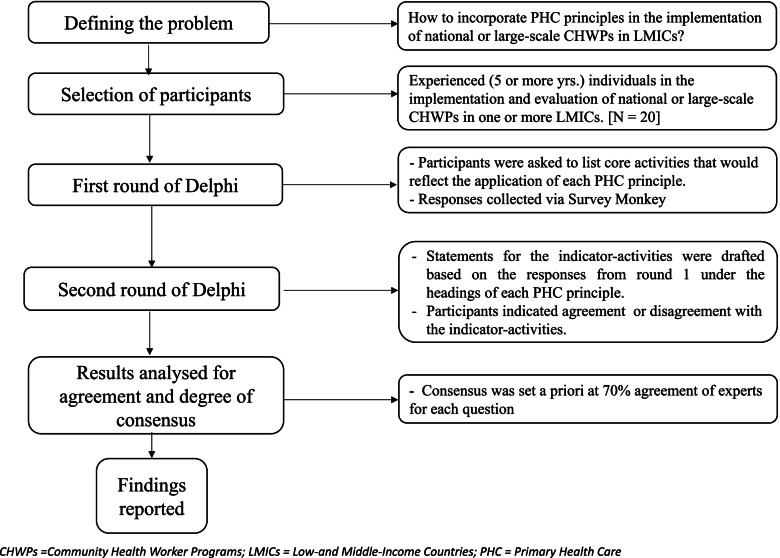
Step-wise process for undertaking Delphi survey

Participants’ responses were collected using a secure online survey program (survey monkey). For round one, participants accessed the survey by a link provided in the email and were required to agree to a statement of consent before commencing the survey. For round two, a separate survey link was provided by email to the study participants. Participants were given two weeks to complete each survey round. One reminder was sent at the end of the first week to maximise the number of responses. The round one survey was closed to allow analysis before the opening of the second survey round. Each survey round questionnaire took approximately 20–30 min to complete. Figure 1 outlines the step-wise process for undertaking this Delphi survey.

### Data analysis

An analysis of responses was performed at the completion of each survey round and before the final analysis was undertaken. For the qualitative data from the first round, thematic content analysis [25] of the open text was used to identify the activities for applying PHC principles in national or large-scale CHWPs in LMICs. Statements for round two were developed based on the common themes which emerged from the round one data analysis. Consensus was set a priori at 70% agreement of experts for each question [21]. Consensus was considered as ‘not met’ if the agreement was < 70% for each question. The list of agreed activities by participants was then synthesised further to develop a set of indicator-activities for each PHC principle and their sub-attributes with examples of types of activities for each indicator-activity.

### Participants and public involvement

The summary results of the Delphi round one have been shared with the participants. Upon publication the final article will also be shared with the participants.

### Positionality statement

Considering our combined work experiences and perspectives, as the authors we acknowledge that there is a possibility that this could impact our analysis and interpretation of the data. Thus, we have been reflexive of our positions and perspectives, and watchful, both individually and collectively, for any potential bias. Reflexive practice has helped us to achieve more objective research, including the design, data collection methods, analysis and interpretations. All authors are researcher-academicians in the field of public health. All authors are currently based in Australia, however one is a Pakistani national and two are Australian nationals, one of whom is of Pakistani origin.

## Results

### Round one

Seventeen of the 20 participants (response rate = 85%) responded to the first survey round. These participants represented a range of professional expertise including program managers, researcher-academics, community engagement advisors, research project managers and advisors for monitoring and evaluation. Their demographics are presented in Table 2 below.

**Table 2:** Participant characteristics (*n* = 17)

**Characteristic**	**Frequency**	**Percentage**
**Country of residence (WHO Regions)**		
*Region of the Americas*	3	17.6
Brazil		
Canada		
Unites States of America		
*African Region*	8	47.1
Ethiopia		
Ghana		
Kenya—3 participants		
Mozambique		
Rwanda		
Zambia		
*South-East Asia Region*	4	23.5
Bangladesh		
India		
Indonesia		
Myanmar		
*Eastern Mediterranean Region*	1	5.9
Pakistan		
*Western Pacific Region*	1	5.9
Philippines		
**Gender**		
Male	8	47.1
Female	9	52.9
**Age**		
< 40 years	5	29.4
> 40 years	12	70.6
**Qualification**		
Doctoral Scientists	9	52.9
Master’s degree	6	35.3
Others	2	11.8
**CHW Program Experience**		
Evaluation and Implementation	11	64.7
Research and Evaluation	2	11.8
Research and Implementation	2	11.8
Research	1	5.9
Others	1	5.9
**Years of Experience**		
5–10 years	8	47.1
10–20 years	6	35.3
20 + years	3	17.6

Consensus was reached on the importance of all the PHC principles in the implementation of national or large-scale CHWP in LMICs. The ranking of these principles in terms of their importance was more difficult for participants; however, consensus was reached on the point that community participation was the most important PHC principle to apply to achieve successful CHWPs in LMICs. Intersectoral coordination was reported as the most challenging PHC principle to implement in round one.

Analysis of open text qualitative data from round one identified the activities reflecting the application of each principle by the national or large-scale CHWPs. Participants also listed a number of challenges involved in applying PHC principles by CHWPs in LMICs.

### Round two

Sixteen participants (response rate = 80%) who initially completed the first survey round completed the second round of the survey. A list of all the activities reported by the participants in round one is presented in Table 3 along with the level of agreement reached in round two of the Delphi exercise. Table 4 illustrates the level of agreement reached among participants for each of the identified challenges that they reported in relation to applying PHC principles in CHWPs.

**Table 3:** Activities and agreement reported by the experts for the implementation of primary health care principles in Delphi rounds one and two

Principles	Activities	Level of agreement (%)
UNIVERSAL HEALTH COVERAGE	Provision of basic maternal, newborn and child health services	93.8
	Medical care services for physical and mental health	93.3
	Appropriate distribution of resources (Staff and material)	87.5
	Defining the catchment area	86.7
	Community sensitization	86.7
	Transparent distribution of resources	86.7
	Outreach services to remote areas	81.3
	Evaluation of the program implementation	69.2
	Annual [re]planning for implementation	57.1
*Equity*	Equity-based planning from the beginning	100
	Identification of groups that are discriminated against	100
	Removing financial and geographic barriers to health care	100
	Implementation focused on vulnerable sub-populations	93.8
	Service packages are adapted to the particular needs of disadvantaged groups	93.8
	Provision of services in hard to reach areas	87.5
	Gender mainstreaming	85.7
	Broadening of selection criteria of CHWs e.g. low literacy groups and women	78.6
	Bottleneck analyses	68.8
	Program cost discussion with the community representatives	50
*Access*	Identification of the causes of low demand and utilization	100
	Ensuring all community members can access the program	100
	Distribution of CHWs across a population	93.8
	Addressing privacy and confidentiality	81.3
	Ensuring financial protection	68.8
	Training and mentorship of CHWs	56.3
	Remuneration arrangements for CHWs in case of emergency	56.3
	Role clarity between the community, CHWs and supervisors/program	50
*Comprehensiveness*	Provision of preventive, curative, and rehabilitative services	100
	Linkages with higher level service providers	87.5
	Needs assessment	81.3
	Referral for and management of endemic illnesses	80
	Skilled CHWs	66.7
	Pro-active CHWs	53.3
COMMUNITY PARTICIPATION	Engaging traditional and other community leaders	100
	Ensuring feedback by the community [and acting on it]	92.9
	Involving community members in supervision of the program activities	87.5
	A practical monitoring system incorporating data from communities and the health system	87.5
	Joint ownership and design of CHW programs	81.3
	Availability of health data to the community	80
	Community sensitization and awareness of the program activities	75
	The integration of CHWs in health care decisions	75
	A balanced package of incentives for CHWs, both financial and non-financial	62.5
INTERSECTORAL COORDINATION	Senior leadership of the program—accessible and flexible	93.8
	CHWs working with community development personal and government officials	93.3
	Addressing needs of water, sanitation, food, housing, transport	87.5
	Horizontal integration at the service delivery level	87.5
	Involvement of multiple ministries/sectors	81.3
	Collaboration in governance structures from local to national level	80
	Partner mapping: to identify all partners who are implementing CHW related interventions	66.7
	Vertical integration within the health systems	46.7
APPROPRIATENESS	Need-based and context specific program design and implementation	93.3
	Prioritization of technically sound and operationally manageable service packages with max health impact	86.7
	Competent CHWs	86.7
	Respectable CHWs	80
	CHW program follows international ethical and human rights standards	66.7
*Effectiveness*	Monitoring to assess outputs with reference to the stated goals	100
	Review of health outcomes and from an equity lens	93.3
	Consistent access to required training, supplies and supervision for CHWs	86.7
	Monitoring and performance systems	80
	Clear coordination	71.4
	Achievement of the target of the specific programs	66.7
*Cultural acceptability*	Community involvement in the selection of the CHWs	100
	CHWs are in high demand, have access to all community members	93.3
	Monitoring to make sure that people understand the messages shared by CHWs	86.7
	Community ownership	85.7
	Community working with CHWs to address needs and concerns in an acceptable way	66.7
	Situation analysis of the target population	64.3
	Relevance of the primary health care, MNCH and reproductive health services	60
*Affordability*	Financial assessment of chosen intervention to envision sustainability	86.7
	Assess if transport cost is a barrier and provide subsidy/transport	86.7
	Assess the ability of the local community to pay	80
	Identify the costs of alternate interventions	78.6
	Assess if the full spectrum of treatment needed is affordable	73.3
	Provision of a basic package of health services that are cost effective	66.7
	Drugs dispensed free to all people irrespective of their ability to pay	53.3
*Manageability*	Adequate human resource	92.9
	Regular provision of a comprehensive package of services at a high standard of quality to all in need	86.7
	Adequate supportive supervision and performance review	85.7
	Continuous adjustment of the role of CHWs as the program evolves with respect to communities’ needs	85.7
	A balanced package of financial and non-financial incentives for CHWs	66.7
	Majority of people are provided the needed services at the cost they can afford	66.7

**Table 4:** Challenges to implement primary health care principles in community health worker programs

CHALLENGES	Level of agreement
Poor leadership and Governance	93.3
Inadequate resource allocation	93.3
Poor understanding of community needs	92.9
Sustainable funding	86.7
Geographic location	80
Political commitment	80
Intersectoral collaboration	80
Inadequate human resource for health	80
Understanding of PHC by the senior decision makers	80
Top-down approach	80
Adopting national approaches with flexible context-specific strategies	78.6
Non-involvement of critical stakeholders in non-health sectors	73.3
Misunderstanding of role of CHW as "doctor"	53.3
Taking CHW programs outside the bio-medical framework	50

Based on participant responses for the activities that reached consensus (Table 3), a set of 29 PHC indicator-activities for the four PHC principles, 1) UHC; 2) community participation; 3) intersectoral coordination; and 4) appropriateness; and their subsequent sub-attributes was developed with examples of types of activities for each indicator-activity (Table 5).

**Table 5:** Indicator activities to implement PHC principles in national or large-scale community health worker programs

PHC Principle	Indicator Activity	Examples of the activity
**UNIVERSAL HEALTH COVERAGE**	Service Provision	•Provide maternal, newborn and child health services •Provide medical care services for physical and mental health •Provide outreach services to remote areas •Horizontal integration at the service delivery level
	Selection and placement of CHWs	•Select CHWs based on a broad criteria not limited by a literacy threshold •Have CHWs in all areas of the country, even the remotest hamlets •Distribute CHWs across a population to make it feasible for the CHW workload and individual care seeking
	Defined catchment area	•Define the catchment area with reference to the population that is to be served by the CHW program. This would facilitate needs assessment, service provision and connection to the formal health system in an organised manner
	Community Sensitization	•Inform the community about the core activities of the coverage •Ensure the community is aware of their right to have access to the needed care
	Needs assessment	•Identify varying needs of sub-population groups to provide equity-based care •Assess the staff and material needs of sub-population to distribute them accordingly •Assess what could work or not in each community in a manner (sensitive to social, economic and cultural aspects) and with a social determinants of health lens – Comprehensiveness
*Equity*	Planning	•Plan services that address the local inequities in service coverage and health outcomes across different types of demographics •Plan services with an understanding about dynamics of discrimination within the local context
	Implementation	•Provide services according to the needs of disadvantaged groups
	Address financial and geographic barriers to health care	•No user fee especially in rural health centres •Provide PHC services close to the community through outreach
*Access*	Identification of the causes of low demand and utilization	•Identify physical barriers and other supply-based barriers like access to quality care and human resources for health, supplies and commodities
	Promote community access to the program	•Ensure that all community members can access the program irrespective of distance, ethnic or religious group, gender, age, social status, physical and mental state, and ability to pay
	Ensure privacy and confidentiality	•Train CHWs to provide services considering privacy and confidentiality of the community members
*Comprehensiveness*	Provision of health services along the spectrum of preventive, curative, and rehabilitative services	•Presence of a functional health unit within the catchment area with primary health care activities
	Linkages with secondary and tertiary level services	•Establish linkages with other service providers and referral pathways to ensure comprehensiveness of a service package, especially if very few or no curative services are being provided directly by the CHWs •Collaborate in governance structures from local to national level
**COMMUNITY PARTICIAPTION**	Joint ownership and design of CHWPs	•Engage community representatives to make sure that they are aware and involved in the design, implementation and evaluation of the program •Involve community at all levels of decision making from planning, training, selecting and oversight of CHWs •Ensure feedback from the community
	Availability of health data to the community	•Ensure that the community is informed, provide feedback and participate in decision-making •Establish a practical monitoring system incorporating data from communities and the health system
**INTERSECTORAL COORDINATION**	Representation of non-health organisations on planning and governance structures of CHWPs	•Negotiate to promote health and addressing needs of water, sanitation, food, housing and transport
	Public private partnership	•CHW program works with [other actors] in the community development sector •CHW program works with government officials •Provide benefit packages to particular populations (e.g. cash transfers for pregnant and lactating woman or households below the poverty line)
**APPROPRIATENESS**	Context specific program design and implementation	•Plan and implement interventions which adhere to community culture and demand
	Evidence-based interventions	Prioritize technically sound and operationally manageable service packages with maximum health impact
*Effectiveness*	Monitoring health outcomes	•Assess health outcomes with reference to the stated goals and from an equity lens •Ensure that quality of care is an integral part of the monitoring systems
	Monitoring performance	•Assess the competence of CHWs regularly on to make sure that they are skilled to address poor health and confident to be pro-active in using these skills
	Well-resourced CHWs	•Provide regular training, supplies and supervision to CHWs in order to ensure intended health outcomes
*Cultural acceptability*	Community involvement in the selection of the CHWs	•Consider factors influencing care-seeking by underserved groups e.g. language and other cultural norms
	Health Literacy	•Monitor that messages shared by CHW program [are such] to which people [relate to] and understand
*Affordability*	Cost effective interventions	•Assess the chosen and alternate interventions financially and in a context-specific manner •Assess if the full spectrum of treatment needed is affordable by the CHW program
	Identify and address financial barriers to health care	•Assess if transport cost is a barrier and provide subsidy/transport if necessary
*Manageability*	Adequate human resources	•Supervisors, program managers and frontline health staff must have the capacity, clear role, time and resources to provide adequate supportive supervision and performance review
	Proportionate service provision	•Consider the range and complexity of services along with the size of the population to be served
	Continuous adjustment of the role of CHWs as the program evolves with respect to communities’ needs	•Full-time, salaried CHW versus part-time, voluntary CHW •Make sure that the time commitment and renumeration of the CHWs are according to service package and catchment area

#### PHC Indicator-Activities for Universal Health Coverage

Five overarching indicator-activities for the principle of UHC were identified along with eight indicator-activities for the sub-attributes of ‘equity’, ‘access’ and ‘comprehensiveness’. In the application of UHC, the indicator- activities encompass: service provision such as provision of medical care, outreach services and targeted services such as maternal and child care; defined catchment areas for the population being served; needs assessments being undertaken to ensure services meet community needs; appropriate selection of placement for CHWs; and community sensitisation where programs undertake activities that inform the community of services and their rights to care. The sub-attribute indicator-activities for ‘equity’ are planning and implementation for the provision of services according to need and taking into account the financial and geographical barriers to such services. As one of the participants highlighted:

“Understanding inequities in service coverage and health outcomes across different types of demographics as well as dynamics of discrimination within the local context is indeed important. Service delivery approaches can and should be tailored and planned with these understandings in mind. Community Health Programs should contribute to building inclusive health systems for people of all abilities, gender identities, ethnicities, etc.” (Participant 4).

The sub-attribute indicator-activities for ‘access’ include identification of cause for low demand, promotion of the program to the community and maintaining privacy and confidentiality. While the sub-attribute indicator-activities for ‘comprehensiveness’ include activities to provide a breadth of services and linkages with secondary and tertiary care.

#### PHC Indicator-Activities for Community participation

Two PHC indicator-activities were identified for community participation encompassing joint ownership and design of the CHWPs and availability of health data to the community. Joint ownership and design of the CHWPs include: identification of community leaders and representatives; engaging them in the design, implementation and evaluation of the CHWPs; and involving community at all levels from planning, selecting, training and oversight of CHWs. Availability of health data to the community facilitates community feedback and contributes to the establishment of a practical monitoring system which can incorporate data from communities and the health system. As one participant noted:

“Data should indeed be available to communities in order for them to be informed, provide feedback and participate in decision-making etc., but making the data available alone does not indicate community participation” (Participant 4).

#### PHC Indicator-Activities for Intersectoral Coordination

For the application of intersectoral coordination, the indicator-activities need to have non-health organisations represented in the planning and governance structures of CHWPs, in order to engage different sectors in the promotion of health, in particular to address the basic needs for water, sanitation, food, housing and transport. Another indicator-activity which reflects intersectoral coordination is public private partnership which requires CHWPs to engage with other actors in the community development sector and with government officials. This would then facilitate access to services and resources that are required for community needs beyond their health care needs. Multiple sectors thus need to collaborate to create supporting approaches to both the renumeration and career opportunities for the CHWs, and also to the provision of packages that would benefit particular populations such as cash transfers for pregnant and/or lactating women or to households living below the poverty line. As indicated by one of the study participants:

“When all sectors understand their role in supporting health and well-being of the people, their actions are synergistic and implement their activities as horizontal programs and not as silo programs” (Participant 13).

#### PHC Indicator-Activities for Appropriateness

Two overarching PHC indicator-activities were identified for the principle of appropriateness along with 10 indicators for the sub-attributes of ‘effectiveness’, ‘cultural acceptability’, ‘affordability’ and ‘manageability’. In the application of appropriateness, the indicator-activities encompass context specific program designs and the implementation and selection of evidence-based interventions adhering to community culture and demand. Prioritization is needed for service packages that consider interventions that are technically sound, operationally manageable and offer the maximum health impact. The sub-attribute indicator-activities for ‘effectiveness*’* include monitoring health outcomes with reference to the stated goals and with equity in mind; well-resourced CHWs with consistent access to required training, supplies and supervision for CHWs to implement CHWPs as designed and in accordance with the expectation of communities; and being able to assess the competence of CHWs. The sub-attribute indicator-activities for ‘cultural acceptability’ are based around community involvement in the selection of CHWs and health literacy of the community achieved through monitoring the messages shared by CHWPs that people both relate to and understand. One of the participants pointed out that:

“Cultural acceptability is met when those who are defined as the objective of an intervention become the subjects and work with CHWs to address both needs and concerns in a way that is acceptable [by the community in the given context]” (Participant 9).

Indicator-activities for the sub-attribute of ‘affordability’ include; the provision of cost-effective interventions such as context-specific cost estimation for chosen and alternate interventions to assess sustainability; and identify and address financial barriers to health care. As one of the study participants highlighted:

“It is important to look at financial barriers (including transport) and cost effectiveness of interventions as well as compare the costs of alternative interventions (i.e., alternative methods for service delivery), but it does not necessarily mean all drugs/services need to be dispensed ‘free of charge’ (though it should be noted that health financing evidence demonstrates that pre-payment and adequate risk-pooling reduces financial barriers)” (Participant 4).

Indicator-activities for the sub-attribute of ‘manageability’ include adequate human resources; proportionate service provision considering the range and complexity of services and the size of the population to be served; and continuous adjustment of the role of CHWs as the program evolves over time with respect to communities’ needs.

#### Challenges in the application of PHC principles by CHWPs

The study participants also reported and agreed on a number of challenges involved in applying PHC principles by CHWPs. A consensus was reached by these participants around the issues of poor leadership and governance with insufficient political commitment and inadequate resource allocation. Other key challenges in applying PHC principles by CHWPs included the lack of adoption of national approaches, difficult geographic locations and poor intersectoral coordination. Study participants highlighted the need for the incorporation of PHC as the main strategy for health services implementation.

One of the participants stated that:

“Some countries have very fragmented health systems – a unified health system makes the application of PHC principles more feasible. Contexts of marked social inequalities are especially challenging” (Participant 6).

Many countries, including LMICs, are renowned for their governments’ top-down approaches or one-way decision-making which ignores the voices of the community. Although some health problems do require strong coordination and government leadership, community engagement is essential for sustainable application of PHC principles.

## Discussion

The study findings demonstrate that experts agreed on 59 core activities which were then used to identify a set of indicator-activities to reflect the application of the PHC principles by CHWPs in LMICs. These indicator-activities provide guidance on how PHC principles can be implemented by CHWPs and be used in the development of new CHWPs as well as assist in their evaluation. The indicator-activities can also be used as a guide to address challenges identified by the study participants in the application of PHC principles by CHWPs.

### Designing new CHWPs

The PHC indicator-activities can be used in the design of new CHWPs to ensure that the principles are applied and maximise the benefits of the CHWP for the community. They can also help guide prioritisation of the area of activities in relation to PHC principles. CHWP policies need to incorporate strategies to implement PHC principles which in turn need to be translated into specific actions and activities at an operational level. To ensure effectiveness when designing CHWPs, it is necessary to begin with a clear understanding of the PHC principles and the indicators which reflect their application on the ground. Careful operational planning based on PHC principles is more likely to result in improved health outcomes at the community level [26].

### Improving CHWP implementation

Evidence suggests that application of PHC principles leads to improved health outcomes [27]; therefore, it is important that CHWPs apply these principles during their implementation. The indicator-activities suggested in this study may contribute towards improving CHWPs (current and existing) by providing guidance on how PHC principles can be applied for better health outcomes. They also provide examples of how the less used principles in CHWPs such as ‘intersectoral collaboration’ could be included. For example, before any implementation, determining how multiple sectors will communicate and interact during the initial planning and funding stages and then after implementation and the evaluation stage of the CHWPs has the potential to facilitate the application of intersectoral coordination and ease the challenges that often accompany the implementation of this principle [28].

### Evaluating CHWPs against PHC

A set of PHC indicator-activities can also be used to evaluate the performance of CHWPs in their application of PHC principles and identify areas of improvement, especially in the presence of a significant dearth of evidence in the evaluation of large-scale or national level CHWPs in LMICs [29–33]. Furthermore, the available evidence on current evaluations of CHWPs, focuses more on the outcomes and process measures related to the program and less on the underlying principles of the program [17]. Therefore, the indicator-activities could be considered as a first step towards adapting a principle-oriented approach for CHWP evaluation. Moreover, there is a lack of standardised measures to assess CHWPs in LMICs [34, 35]. Therefore, data cannot be aggregated across programs/regions, and this also hampers any cross-country comparisons. The indicator-activities identified through this study could thus allow comparisons across CHWPs (national and international) through the use of a common set of indicator-activities [35].

There are existing strategy, function and process oriented frameworks about CHWs and the programs utilising the services of CHWs [13–18]. Some of these existing frameworks do refer to one or two PHC principles, particularly community involvement [17]. The indicator-activities identified in this study through the Delphi exercise explain in some detail that if the CHWPs are aligned with all four fundamental PHC principles and their sub-attributes (Table 1). Moreover, this study emphasises that the direction of the strategy, function and processes of the CHWPs should be based on the principles of PHC.

The results of this Delphi exercise also reaffirm the strategic and operational levers put forward in the WHO Operational framework for translating the global commitments for PHC into actions and interventions [20]. This is a high level framework which mainly targets government leaders in order to accelerate national implementation efforts on PHC. The indicator-activities identified in this Delphi exercise align with the majority of strategic and operational levers in the WHO framework. Therefore, this study is also pertinent to recent guidelines set by the WHO and applied to national or large-scale CHWPs in LMICs. For example, the engagement of community and other stakeholders is one of the four core strategic levers included in the WHO framework which aims to strengthen national health systems. This complements the findings of the Delphi exercise where the participants agreed on a number of key points, importantly including the need to engage community leaders and ensure feedback by the community as well as to establish practical monitoring systems, which feature as one of the operational levers of the WHO framework.

### Strengths and limitations

Use of the modified Delphi approach in this study enabled a pragmatic exploration of the activities to reflect the application of PHC principles in national or large-scale CHWPs in LMICs. Purposive sampling enabled recruitment of participants from different countries, health systems and program development, implementation and evaluation perspectives and improved content validity. Responses from round one, and levels of consensus for each PHC principle, were provided back to the participants for the purpose of the round two of the modified Delphi survey. Each opinion was given the same degree of importance in the analysis in order to eliminate the participant bias [36].

One of the limitations was sample size, although our sample is in line with previously published Delphi survey recommendations [37]. The richness of comments and perspectives shared by participants in the first survey round suggest that this sampling approach was appropriate. Secondly, the Delphi method has potential limitations as participants’ responses might not be truly independent and may not be generalizable to settings of which the participants may not have any experience [38]. Anonymity between the study participants enabled participants to be open and honest about their views as well as providing them with an equal opportunity to express an opinion without feeling pressured to conform to the views of others [36].

## Conclusion

This study has identified 29 core indicator-activities which can provide guidance on how PHC principles can be implemented in CHWPs in LMICs. These indicator-activities can be used in the development of new CHWPs and assist in the evaluation of CHWPs, particularly in their application of PHC principles. Future research may focus on testing the utility and applicability of PHC indicator-activities on CHWPs and involving more stakeholders such as CHWs themselves.

## Supplementary Information

**Additional file 1.** Primary Health Care Principles and Community HealthWorker Programs in Low and Middle Income Countries(DELPHI ROUND ONE).

**Additional file 2. **Primary Health Care Principles and Community HealthWorker Programs in Low and Middle Income Countries(DELPHI ROUND TWO).

## Data Availability

All data relevant to the study are included in the article.

## Abbreviations

CHWP: Community Health Worker Programs
LMICs: Low-and Middle-Income Countries
PHC: Primary Health Care
WHO: World Health Organisation
