# Supplementary material for: Indicator-activities to apply primary health care principles in national or large-scale community health worker programs in low-and middle-income countries: a Delphi exercise
Source: BMC Public Health. 2022 Aug 22;22:1599. doi: 10.1186/s12889-022-13996-y (PMC9396777; doi:10.1186/s12889-022-13996-y)
Supplement: Supplementary file 1 — Additional file 1. Primary Health Care Principles and Community HealthWorker Programs in Low and Middle Income Countries(DELPHI ROUND ONE). [file 12889_2022_13996_MOESM1_ESM.pdf]

Primary Health Care Principles and Community Health Worker Programs in Low and Middle Income Countries (DELPHI ROUND ONE)

Research Team
Dr Shagufta Perveen, Dr Mohammad Afzal Mahmood, Prof Caroline Laurence
School of Public Health, University of Adelaide, South Australia

Ethics Approval Number: H-2020-179

ABOUT THIS SURVEY!

This survey which is being conducted by researchers at the University of Adelaide, intends to develop consensus regarding implementation of Primary Health Care (PHC) principles in national or large-scale Community Health Worker (CHW) programs in Low-and Middle-Income Countries (LMICs).

This survey will take you approximately 40-60 minutes to complete, and is anonymous to maintain confidentiality.

There are three sections in this survey, please complete each section before moving to the next.

The questions with an asterisk (*) need a mandatory response.

Your contribution to this survey is greatly appreciated.

Primary Health Care Principles and Community Health Worker Programs in Low and Middle Income Countries (DELPHI ROUND ONE)

ELECTRONIC CONSENT

Clicking on the "agree" button below acknowledges that;

- you have read the **Participant Information Sheet** and understand the purpose of the project.
- you understand that participation is voluntary with no direct benefits to you.
- you understand that, while information gained during the study may be published, you will not be identified and your personal responses will not be divulged.
- you understand that, you are free to withdraw from the project at any time.
- you understand that if you have any additional questions or concerns you can contact the research team.
- you are at least 18 years of age.
- Agree to participate in the project.
- Click "Next" to start round one of the survey

* **1.** Do you agree to the above terms? By clicking "Yes", you consent that you are willing to answer the questions in this survey

◯ **Yes, I agree**

◯ **No, I disagree**

Primary Health Care Principles and Community Health Worker Programs in Low and Middle Income Countries
(DELPHI ROUND ONE)

Project Background:

This research project is about developing expert consensus on the relative importance of primary health care (PHC) principles and identify activities to reflect implementation of these principles in national community health worker (CHW) programs in low and middle income countries (LMICs). The aim of this project is to develop guidelines on the application of PHC principles in the implementation of national or large-scale CHW programs in LMICs. The objectives of this research project are;

- To determine the relative importance of PHC principles
- To identify the activities reflecting PHC principles in the implementation of national CHW programs in LMICs
- To identify any additional PHC principle/s related to implementation of CHW programs in LMICs

Bryant in 1988 highlighted the fact that "PHC is the key to achieve 'Health for All' and should include the following five key concepts;

1. Universal coverage on the basis of need: This is the call of equity. Universal coverage aims at that no one should be left out, no matter how poor or how remote. Care according to need (equity) adds a dimension considering that not all have equal needs and for that reason while everyone needs to be served, the extent and level of service needs adjusting according the relative needs of people. Within a context of relative resource scarcity, those most in need should have priority. Here is the All in Health for All.

2. Comprehensiveness: Services should be promotive, preventive, curative and rehabilitative. Services should not only be curative, but should also be focused at prevention of diseases and promotion of health through education, better environment and healthy policies. The services need to target the root causes of disease with preventive emphasis. Treatment of illness and rehabilitation are important as well.

3. Community Participation: The community's role must be more than that of responding to services planned and designed from the outside. The community should be actively involved in the entire process of defining health problems and needs, developing solutions and implementing and evaluating programs.

4. Inter-sectoral Coordination: A larger spectrum of social determinants determine health. Hence, factors that enhance or reduce health also reside in domains that relate to other sectors which have a substantial impact on health in particular education, clean water and sanitation, agriculture, improved housing and infrastructure of transportation, communication, safety and security, rights of sub-populations groups and minorities etc.

5. Appropriateness: Services should be effective, culturally acceptable, affordable and manageable. Effectiveness (in terms of potential to achieve intended health outcomes) and acceptability (from cultural and social perspectives) are mutually dependent. Services must also be affordable to individuals and implementable within the context of local economic development with a consideration to available governmental resources and becausethe community will often have to share in the costs.

The process of implementing PHC principles however, has been challenging. The Alma Ata declaration proposed the development of national CHW programs as an important policy for promoting PHC and achieving better health outcomes. National CHW programs were created by many governments as a popular way to incorporate PHC principles into health policies and programs. However, several gaps are evident in the existing literature reflecting on the limited application of these principles in the national or large-scale CHW proms in the context of LMICs. Therefore, this research project aims to develop guidelines on the application of PHC principles in the implementation of national or large-scale CHW programs in LMICs.

Operational Definitions:

1. A national CHW program is one that is authorized and supported by the national health system and is deployed nationally.

2. A large-scale program is one that involves a large number of CHWs but is not a government-sponsored program.

3. The concept of Universal Health Coverage (UHC) now incorporates the concepts of universal coverage and comprehensiveness therefore, this research focuses on the four PHC principles; (i) UHC (ii) Commuity participation (iii) Intersectoral coordination and (iv) Appropriateness.

Primary Health Care Principles and Community Health Worker Programs in Low and Middle Income Countries

(DELPHI ROUND ONE)

SECTION 1: DEMOGRAPHICS

The following questions are about yourself:

* 2. In what country do you currently reside? (please select from the dropdown options)

3. What is your gender?

- ◯ Female
- ◯ Male

4. What is your age?

* 5. Your Qualification(s)?

6. Does your current job relate to community health workers OR CHW Program(s)?

- ☐ Yes
- ☐ No
- ☐ Other (please specify)

* 7. Your current role/ job title?

*** 8. Have you been involved in the evaluation/s of national CHW programs in LMICs?**

- ☐ Yes
- ☐ No

*** 9. Have you been involved in the implementation of national CHW programs in LMICs?**

- ☐ Yes
- ☐ No

*** 10. Approximate number of years of experience in planning, providing advice on planning and/or implementation and/or evaluation of national CHW programs in LMICs?**

- ◯ less than 5 years
- ◯ 5-10 years
- ◯ 10-15 years
- ◯ 15-20 years
- ◯ more than 20 years

Primary Health Care Principles and Community Health Worker Programs in Low and Middle Income Countries

(DELPHI ROUND ONE)

SECTION 2: PRIMARY HEALTH CARE PRINCIPLES

In this section you will be asked about the importance of PHC principles and the challenges in their implementation.

11. Please rate the importance of incorporating the PHC principles (universal health coverage, community participation, intersectoral coordination, appropriateness) in the implementation of national CHW programs in LMICs.

Very Important	Important	Somewhat important	Not so important	Not at all important
○	○	○	○	○

12. Explain briefly your reasons to the above rating, relevance of these principles to CHW programs and impact of these principles on implementation and outcomes of the program:

13. Please rank each of the following four PHC principles in terms of its importance in the implementation of national CHW programs in LMICs?

	1 = Most important	2	3	4 = Least important
Universal Health Coverage	○	○	○	○
Community Participation	○	○	○	○
Intersectoral coordination	○	○	○	○
Appropriateness	○	○	○	○

14. Explain briefly your reasons to the above ranking in question 13

15. Are there any PHC principle/s in addition to the four listed above which are relevant to apply in national CHW programs in LMICs?

*** 16. Which PHC principle/s is/are most challenging to implement in national or large-scale CHW programs in LMICs? (please select all that apply)**

- [ ] Universal Health Coverage
- [ ] Community Participation
- [ ] Intersectoral Coordination
- [ ] Appropriateness

17. Explain briefly your reasons for your choice/s in question 16.

Primary Health Care Principles and Community Health Worker Programs in Low and Middle Income Countries

(DELPHI ROUND ONE)

SECTION 3: ACTIVITIES TO IMPLEMENT PRIMARY HEALTH CARE PRINCIPLES

18. In your opinion, what are the activities which reflect the implementation of **'Universal Health Coverage'** in the national or large-scale CHW programs in LMICs?

19. In your opinion, what are the activities which reflect the implementation of **equity** [a sub-attribute of universal health coverage] in the national or large-scale CHW programs in LMICs?

20. In your opinion, what are the activities which reflect the implementation of **access** [a sub-attribute of universal health coverage] in the national or large-scale CHW programs in LMICs?

21. In your opinion, what are the activities which reflect the implementation of **comprehensiveness** [a sub-attribute of universal health coverage] in the national or large-scale CHW programs in LMICs?

22. In your opinion, what are the activities which reflect the implementation of **'Community Participation'** in the national or large-scale CHW programs in LMICs?

23. In your opinion, what are the activities which reflect the implementation of **'Intersectoral Coordination'** in the national or large-scale CHW programs in LMICs?

24. In your opinion, what are the activities/characteristics or features of a program which reflect the implementation of the principle of **'Appropriateness'** in the national or large-scale CHW programs in LMICs?

25. In your opinion, what are the core activities which reflect **effectiveness** [a sub-attribute of the principle of appropriateness] in the national or large-scale CHW programs in LMICs?

26. In your opinion, which characteristics and /or features of a program reflect **cultural acceptability** [a sub-attribute of the principle of appropriateness] in the national or large-scale CHW programs in LMICs?

27. In your opinion, which characteristics and /or features of a program reflect affordability [a sub-attribute of the principle of appropriateness] in the national or large-scale CHW programs in LMICs?

28. In your opinion, which characteristics and /or features of a program reflect that the services are manageable [a sub-attribute of the principle of appropriateness] in the national or large-scale CHW programs in LMICs?

29. In your opinion, what are the challenges to apply PHC principles in the national or large-scale CHW programs in LMICs?

Primary Health Care Principles and Community Health Worker Programs in Low and Middle Income Countries
(DELPHI ROUND ONE)

Thank you very much for taking the time to complete our survey. You contribution to this research is much appreciated!

If you have any queries or further interest in this research, please contact:

Dr Shagufta Perveen
School of Public Health, The University of Adelaide
shagufta.perveen@adelaide.edu.au

END OF SURVEY

Primary Health Care Principles and Community Health Worker Programs in Low and Middle Income Countries
(DELPHI ROUND ONE)

Thank you very much.

If you have any comments or further interest in this research, please contact:

Dr Shagufta Perveen
School of Public Health, The University of Adelaide
shagufta.perveen@adelaide.edu.au

END OF SURVEY
