# Supplementary material for: Indicator-activities to apply primary health care principles in national or large-scale community health worker programs in low-and middle-income countries: a Delphi exercise
Source: BMC Public Health. 2022 Aug 22;22:1599. doi: 10.1186/s12889-022-13996-y (PMC9396777; doi:10.1186/s12889-022-13996-y)
Supplement: Supplementary file 2 — Additional file 2. Primary Health Care Principles and Community HealthWorker Programs in Low and Middle Income Countries(DELPHI ROUND TWO). [file 12889_2022_13996_MOESM2_ESM.pdf]

Primary Health Care Principles and Community Health Worker Programs in Low and Middle Income Countries (DELPHI ROUND TWO)

Research Team
Dr Shagufta Perveen, Dr Mohammad Afzal Mahmood, Prof Caroline Laurence
School of Public Health, University of Adelaide, South Australia

Ethics Approval Number: H-2020-179

ABOUT THIS SURVEY!

This survey which is being conducted by researchers at the University of Adelaide, intends to develop consensus regarding implementation of Primary Health Care (PHC) principles in national or large-scale Community Health Worker (CHW) programs in Low- and Middle-Income Countries (LMICs).

The purpose of round two of the Delphi study is to develop consensus on the narrative responses received in round one from the study participants.

This survey will take you approximately 15-30 minutes to complete, and is anonymous to maintain confidentiality.

There are two sections in this survey, please complete each section before moving to the next.

All the questions are mandatory.

Your contribution to this survey is greatly appreciated.

Primary Health Care Principles and Community Health Worker Programs in Low and Middle Income Countries (DELPHI ROUND TWO)

CONSENT REMINDER

By clicking on the "NEXT" button below you are indicating that;

- you have read the **Participant Information Sheet** and understand the purpose of the project.
- you understand that participation in this research project is entirely voluntary with no direct benefits to you.
- you understand that, while information gained during the study may be published, you will not be identified and your personal responses will not be divulged.
- you understand that, you are free to withdraw from the project at any time.
- you understand that if you have any additional questions or concerns you can contact the research team.
- you are at least 18 years of age.
- Agree to participate in the project.
- Click "Next" to start Round 2 of the survey

THE UNIVERSITY of ADELAIDE

Primary Health Care Principles and Community Health Worker Programs in Low and Middle Income Countries (DELPHI ROUND TWO)

Project Background:

This research project is about developing expert consensus on the relative importance of PHC principles and identify activities to reflect implementation of these principles in national CHW programs in LMICs.

The aim of this project is to develop guidelines on the application of PHC principles in the implementation of national or large-scale CHW programs in LMICs.

Primary Health Care Principles and Community Health Worker Programs in Low and Middle Income Countries (DELPHI ROUND TWO)

SECTION 1: PRIMARY HEALTH CARE PRINCIPLES - Importance and challenges in implementation

- In the Round 1 Survey, all the participants rated PHC principles as very important or important to incorporate in the implementation of national or large-scale CHW programs in LMICs - consensus reached

- For the ranking of PHC principles in terms of importance in the implementation, we reached consensus on the principles of "Community Participation" and "Intersectoral Coordination". However, consensus was not reached on the importance of "Universal Health Coverage" and "Appropriateness".

* **1.** Therefore, please rate which of these two PHC principle is relatively more important than the other in relation to national or large-scale CHW programs in LMICs (select only one).

☐ **Universal Health Coverage**

☐ **Appropriateness**

* **2.** From the Round 1 Survey, participants reported a number of additional PHC principles. Please select which of the following additional principles you agree should be added to the original PHC principles.

	AGREE	DISAGREE
**Political Commitment**	○	○
**Sustainability**	○	○
**Social Justice**	○	○
**Accountability to the community**	○	○
**Appropriate (or Good) leadership and governance**	○	○

3. In the Round 1 Survey, we reached consensus on that the principles of "Community Participation" and "Appropriateness" were challenging to implement. However, consensus was not reached for other principles.
For the following principles please determine which one do you consider as MORE CHALLENGING than the other to implement in CHW programs in LMICs (select only one).

- ☐ **Universal Health Coverage**
- ☐ **Intersectoral Coordination**

Primary Health Care Principles and Community Health Worker Programs in Low and Middle Income Countries (DELPHI ROUND TWO)

SECTION 2: ACTIVITIES TO IMPLEMENT PRIMARY HEALTH CARE PRINCIPLES

- We have summarized the activities reported by the participants in Round 1 of the Survey that reflect the implementation of each PHC principle and its sub-attributes.

- For each list of activities reported for the PHC principles and its sub-attributes, please indicate if you AGREE or DISAGREE that the suggested activity reflects the PHC principle by ticking the appropriate boxes.

UNIVERSAL HEALTH COVERAGE (UHC):

* **4.** Please indicate if you AGREE or DISAGREE that the suggested activity indicates **UHC** by ticking the appropriate boxes.

	AGREE	DISAGREE
**Defining the catchment area (with reference to the population that is to be served by the CHW program)**	○	○
**Community sensitization: informing the community about services that will be provided**	○	○
**Annual [re]planning for implementation**	○	○

	AGREE	DISAGREE
Evaluation of the program implementation	◯	◯
Appropriate distribution of resources (Staff and material)	◯	◯
Transparent distribution of resources	◯	◯
Provision of basic maternal, newborn and child health services	◯	◯
Outreach services to remote areas	◯	◯
Medical care services for physical and mental health	◯	◯

5. Comments (if any):

Equity (a sub-attribute of UHC):

* **6.** Please indicate if you AGREE or DISAGREE that the suggested activity indicates **Equity** by ticking the appropriate boxes.

	AGREE	DISAGREE
Equity-based planning from the beginning	◯	◯
Bottleneck analyses	◯	◯

	AGREE	DISAGREE
Identification of groups that are discriminated against	◯	◯
Implementation focused on vulnerable sub-populations	◯	◯
Service packages are adapted to the particular needs of disadvantaged groups	◯	◯
Provision of services in hard to reach areas	◯	◯
Removing financial and geographic barriers to health care	◯	◯
Program cost discussion with the community representatives	◯	◯
Gender mainstreaming	◯	◯
Broadening of selection criteria of CHWs e.g. low literacy groups and women	◯	◯

7. Comments (if any):

Access (a sub-attribute of UHC):

* **8.** Please indicate if you AGREE or DISAGREE that the suggested activity indicates **Access** by ticking the appropriate boxes.

	AGREE	DISAGREE
Identification of the causes of low demand and utilization	○	○
Addressing privacy and confidentiality	○	○
Training and mentorship of CHWs	○	○
Distribution of CHWs across a population	○	○
Role clarity between the community, CHWs and supervisors/program	○	○
Renumeration arrangements for CHWs in case of emergency	○	○
Ensuring financial protection	○	○
Ensuring all community members can access the program irrespective of distance, ethnic or religious group, gender, age, social status, physical and mental state, and ability to pay	○	○

9. Comments (if any):

Comprehensiveness (a sub-attribute of UHC):

* **10.** Please indicate if you AGREE or DISAGREE that the suggested activity indicates **Comprehensiveness** by ticking the appropriate boxes.

	AGREE	DISAGREE
**Provision of preventive, curative, and rehabilitative services**	○	○
**Linkages with higher level service providers, referral pathways and other sectors (social protection, education, etc.)**	○	○
**Referral for and management of endemic illnesses within acceptable distance of nearest health facility**	○	○
**Skilled CHWs to participate in decisions about health care addressing social determinants**	○	○
**Pro-active CHWs to participate in decisions about health care addressing social determinants**	○	○

	AGREE	DISAGREE
**Needs assessment (sensitive to social, economic, cultural aspects) and with a social determinants of health lens**	◯	◯

11. Comments (if any):

Primary Health Care Principles and Community Health Worker Programs in Low and Middle Income Countries (DELPHI ROUND TWO)

COMMUNITY PARTICIPATION:

* **12.** Please indicate if you AGREE or DISAGREE that the suggested activity indicates **Community Participation** by ticking the appropriate boxes.

	AGREE	DISAGREE
Joint ownership and design of CHW programs: Involving community at all levels for decision making from planning, training, selecting and oversight of CHWs, and in local management and monitoring - Key to sustainability	○	○
Availability of health data to the community	○	○
Community sensitization and awareness of the program activities	○	○

	AGREE	DISAGREE
Traditional leaders and other community leaders should be engaged	○	○
Involving community members in supervision of the program activities	○	○
Ensuring feedback by the community [and acting on it]	○	○
A practical monitoring system incorporating data from communities and the health system	○	○
The integration of CHWs in health care decisions that go beyond medical care and address wider issues concerning power and control, advocacy and social mobilization	○	○
A balanced package of incentives for CHWs, both financial and non-financial	○	○

13. Comments (if any):

Primary Health Care Principles and Community Health Worker Programs in Low and Middle Income Countries (DELPHI ROUND TWO)

INTERSECTORAL COORDINATION:

* **14.** Please indicate if you AGREE or DISAGREE that the suggested activity indicates **Intersectoral Coordination** by ticking the appropriate boxes.

	AGREE	DISAGREE
Partner mapping: to identify all partners who are implementing CHW related interventions	○	○
Involvement of multiple ministries/sectors in supporting approaches to renumeration and career ladder opportunities for CHWs	○	○
Collaboration in governance structures from local to national level	○	○
Horizontal integration at the service delivery level	○	○

	AGREE	DISAGREE
Vertical integration within the health systems	◯	◯
Senior leadership requires to be accessible and flexible to negotiate with different sectors to promote health	◯	◯
When CHWs work with community development personal to solve problems beyond service delivery and work with government officials to meet community needs beyond the health sector	◯	◯
Addressing needs of water, sanitation, food, housing, transport	◯	◯

15. Comments (if any):

THE UNIVERSITY *of* ADELAIDE

Primary Health Care Principles and Community Health Worker Programs in Low and Middle Income Countries (DELPHI ROUND TWO)

APPROPRIATENESS:

* **16.** Please indicate if you AGREE or DISAGREE that the suggested activity indicates **Appropriateness** by ticking the appropriate boxes.

	AGREE	DISAGREE
Need-based and context specific program design and implementation	○	○
CHW program follows international ethical and human rights standards	○	○
Competent CHWs	○	○
Respectable CHWs	○	○
Prioritization for service packages should consider interventions that are technically sound, operationally manageable and most promising in their potential for maximum health impact	○	○

17. Comments (if any):

Effectiveness (a sub-attribute of appropriateness):

* **18.** Please indicate if you AGREE or DISAGREE that the suggested activity indicates **Effectiveness** by ticking the appropriate boxes.

	AGREE	DISAGREE
Clear coordination	◯	◯
Monitoring and performance systems	◯	◯
Achievement of the target of the specific programs	◯	◯
Consistent access to required training, supplies and supervision for CHWs to implement program as designed and in accordance with expectation of communities	◯	◯
Review of health outcomes and from an equity lens	◯	◯
Monitoring to assess outputs with reference to the stated PHC, Maternal Newborn Child Health and Reproductive Health goals	◯	◯

19. Comments (if any):

Cultural acceptability (a sub-attribute of appropriateness):

* **20.** Please indicate if you AGREE or DISAGREE that the suggested activity indicates **Cultural Acceptability** by ticking the appropriate boxes.

	AGREE	DISAGREE
Situation analysis of the target population	○	○
Community ownership	○	○
Relevance of the primary health care, MNCH and reproductive health services will help out in assessing acceptability	○	○
Community involvement in the selection of the CHWs	○	○
Monitoring to make sure that people understand the messages shared by CHWs and feel that they belong to the health system	○	○
CHWs are in high demand, have access to all community members, and their advices are welcomed and appreciated	○	○

	AGREE	DISAGREE
Cultural acceptability is met when those who are defined as the objective of an intervention become the subjects and work with CHWs to address both needs and concerns in a way that is acceptable	○	○

21. Comments (if any):

Affordability (a sub-attribute of appropriateness):

* **22.** Please indicate if you AGREE or DISAGREE that the suggested activity indicates **Affordability** by ticking the appropriate boxes.

	AGREE	DISAGREE
Assess the ability of the local community to pay	○	○
Identify the costs of alternate interventions	○	○
Make an assessment / estimation whether chosen interventions are financially viable for longer period of time in that country	○	○
Assess if transport cost is a barrier and provide subsidy/transport	○	○
Assess if the full spectrum of treatment needed is affordable [not only the counselling/primary treatment by CHW]	○	○
Drugs dispensed free to all people irrespective of their ability to pay	○	○
Provision of a basic package of health services that are cost effective	○	○

23. Comments (if any):

Manageability (a sub-attribute of appropriateness):

*** 24.** Please indicate if you AGREE or DISAGREE that the suggested activity informs that the services [of a CHW Program] are **Manageable** by ticking the appropriate boxes.

	AGREE	DISAGREE
**Regular provision of a comprehensive package of services at a high standard of quality to all the individuals in need of the services within their catchment area**	○	○
**A balanced package of financial and non-financial incentives is required for CHWs**	○	○
**Supervisors are available who have capacity, clear role, time and resources for adequate supportive supervision and performance review**	○	○
**Majority of people are provided the needed services at the cost they can afford**	○	○

	AGREE	DISAGREE
A continuous adjustment of the role of CHWs as the program evolves over time with respect to communities' needs	○	○
Providing adequate human resource	○	○

25. Comments (if any):

Primary Health Care Principles and Community Health Worker Programs in Low and Middle Income Countries (DELPHI ROUND TWO)

The following **challenges** have been reported by the study participants in the Round 1 Survey when applying PHC principles in the national or large-scale CHW programs in LMICs.

* **26.** For each of the following challenges, please indicate if you AGREE or DISAGREE that this is a challenge for the implementation of PHC principles in CHW Programs in LMICs.

	AGREE	DISAGREE
Geographic location	◯	◯
Inadequate resource allocation	◯	◯
Sustainable funding	◯	◯
Poor understanding of community needs	◯	◯
Non-involvement of critical stakeholders in non-health sectors	◯	◯
Political commitment	◯	◯
Intersectoral collaboration	◯	◯
Poor leadership and Governance	◯	◯

	AGREE	DISAGREE
Inadequate Human Resource for Health	◯	◯
Misunderstanding of role of CHW as "doctor"	◯	◯
Understanding of PHC by the senior decision makers	◯	◯
Top-down approach where the government tends to make one-way decisions and leave people's voices out	◯	◯
Taking CHW programs outside the bio-medical framework	◯	◯
Adopting national approaches with flexibile context-specific strategies	◯	◯

27. Comments (if any):

Primary Health Care Principles and Community Health Worker Programs in Low and Middle Income Countries (DELPHI ROUND TWO)

Thank you very much for taking the time to complete our survey. You contribution to this research is much appreciated!

If you have any queries or further interest in this research, please contact:

Dr Shagufta Perveen
School of Public Health, The University of Adelaide
shagufta.perveen@adelaide.edu.au

END OF SURVEY
